# Sore Throat: Can It Be Primary Laryngeal Small Cell Carcinoma?

**DOI:** 10.7759/cureus.23327

**Published:** 2022-03-19

**Authors:** Syed Naqvi, Anastasia Schuldt, Amman Yousaf, Shoaib Muhammad, Diego Cabrera

**Affiliations:** 1 Internal Medicine, University of Kentucky College of Medicine, Kentucky, USA; 2 Internal Medicine, McLaren Flint, Flint, USA; 3 Radiology, Salam Medical Complex, Lahore, PAK; 4 Department of Urology, Ghulab Devi Hospital, Al-Aleem Medical College, Lahore, PAK

**Keywords:** metastatic lesion, progressive dysphagia, neuroendocrine neoplasm, small cell carcinomas, laryngeal cancer

## Abstract

Small cell carcinoma of the larynx is a rare form of neuroendocrine carcinoma. Clinical and radiological properties are similar to other laryngeal cancers, prompting histopathology examination. Symptoms include sore throat, dysphagia, odynophagia, and weight loss. Multiple management options have been demonstrated in the literature. However, combined radiation and chemotherapy have proven to improve survival. Unfortunately, the prognosis for this cancer is dismal, as survival from diagnosis rarely exceeds two years. In this article, we present a 64-year-old female patient who presented with a sore throat and was diagnosed with primary small cell cancer of the larynx. Despite the relapse after the initial four cycles of chemotherapy with carboplatin and etoposide, our patient responded well to nivolumab and ipilimumab and is still in remission on a six-month follow-up.

## Introduction

Primary laryngeal carcinoma accounts for approximately 2% to 5% of the global cancer burden. It is more prevalent in males, with a male to female ratio ranging from 2:1 to 4:1. Most of the cases have been reported in the seventh decade and are considered sporadic in younger individuals [1,2]. First reported by Olofsson et al. in 1972, extrapulmonary small cell carcinoma of the larynx is an extremely rare subtype accounting for fewer than 0.5% of all laryngeal malignancies [3]. Smoking, tobacco chewing, and alcohol have been demonstrated among the well-known risk factors. Being an aggressive tumor, it shows early widespread metastasis resulting in a poor prognosis [4]. Small cell laryngeal carcinoma has overlapping clinical manifestations, radiologic features, and the gross appearance of other laryngeal malignancies. Therefore, histopathologic analysis is warranted to confirm the diagnosis. This article presents a primary malignant neuroendocrine tumor of the larynx with metastasis to the liver.

## Case presentation

We present a 64-year-old female who came to our hospital with intermittent sore throat and neck swellings for two months. Her past medical history was significant for chronic obstructive pulmonary disease, essential hypertension, type 2 diabetes mellitus, and cigarette smoking (30 packs year history). The patient had completed multiple antibiotics and analgesics regimens, which temporarily gave her symptomatic relief. However, she started having dysphagia and odynophagia, which warranted further medical care. Interestingly, she also lost significant weight (25 pounds in two months). Initial examination revealed bilateral cervical lymphadenopathy, and laryngoscopy showed ulceration in the false vocal cord and left piriform sinus. 

**Figure 1:**
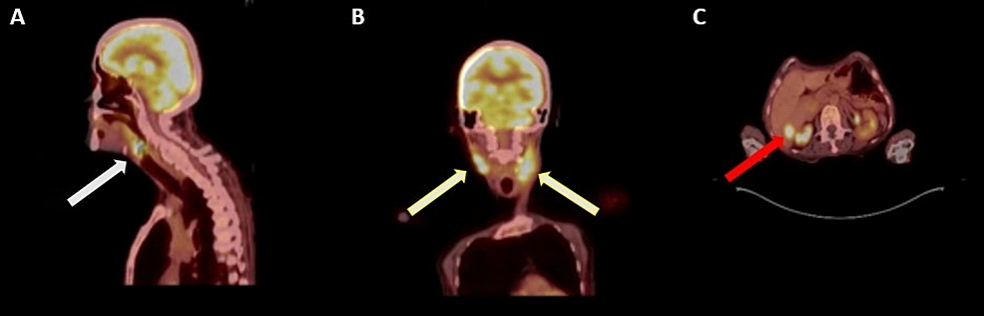
Staging positron emission tomography (PET)/CT (A) It demonstrates primary laryngeal mass (white arrow). (B) There is bilateral hypermetabolic cervical adenopathy (yellow arrow). (C) Section delineates liver metastasis (red arrow).

Biopsy and histopathology from these lesions (laryngeal and lymph nodes) were consistent with poorly differentiated neuroendocrine malignancy. The patient underwent positron emission tomography (PET)/CT for staging, which revealed metastatic lesions in the liver and cervical lymph nodes (Figure 1).

The patient underwent four cycles of chemotherapy (carboplatin and etoposide). 

**Figure 2:**
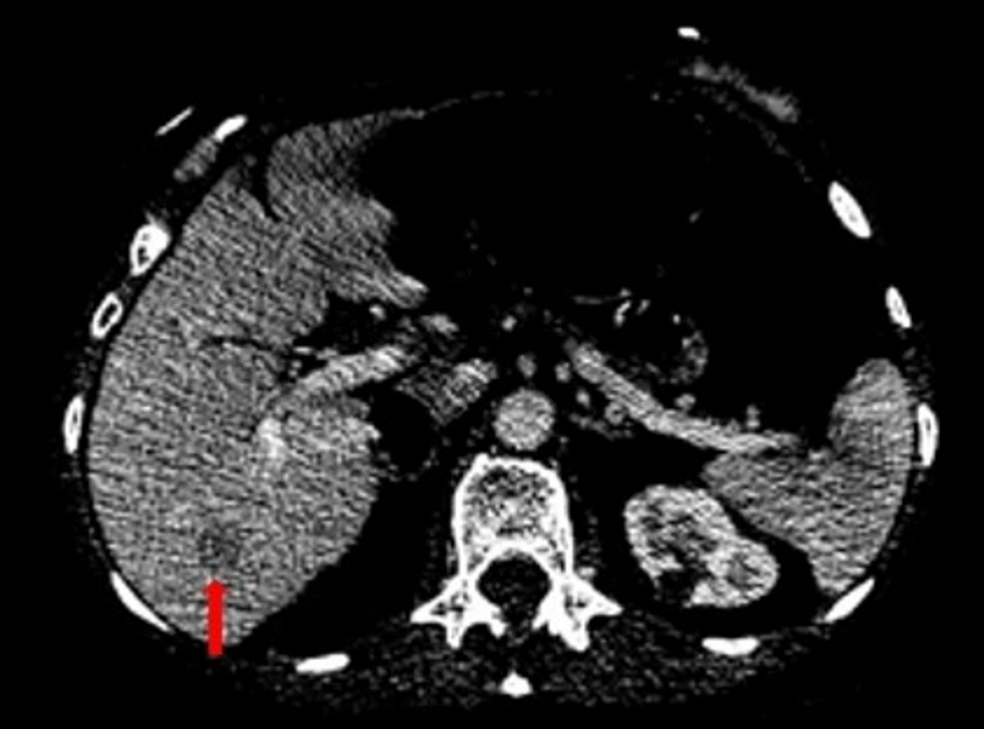
Selected axial section of the CT abdomen demonstrating a hypodense metastatic liver lesion (red arrow).

A follow-up CT scan of the neck, chest, abdomen, and pelvis after three months showed a significant reduction in the size of the primary tumor and lymph nodes size, resulting in improved symptoms. Unfortunately, after three months, the patient's symptoms recurred, and she developed neck swellings associated with dysphagia. A repeat CT neck revealed relapsed disease in her hypopharynx and bilateral cervical nodes. Unfortunately, a staging CT scan revealed an interval increase in the size of the previously seen metastatic lesion in the liver (Figure 2).

The patient was started on combination therapy with nivolumab and ipilimumab and achieved complete resolution. Luckily, no relapse was noted in the repeated imaging and she remained asymptomatic on a six-month follow-up.

## Discussion

The most prevalent laryngeal cancer type is primary squamous cell carcinoma accounting for 99% of the cases. In contrast, neuroendocrine tumors rarely present as primary laryngeal cancers (0.5%). According to the 2017 WHO classification of head and neck cancers, laryngeal neuroendocrine tumors are subdivided into three categories: well-differentiated, moderately differentiated, and poorly differentiated subtype (including small-cell and large-cell neuroendocrine carcinomas) [5,6]. The confirmation of subtype by histological examination and diagnosis is important to determine the appropriate treatment and prognosis, as these vary significantly between the subtypes [7,8]. Typical histopathological findings include nests and sheets of small cells. The arrangements of cells are in a trabecular pattern and display a high level of infiltration. Nuclei have granular chromatin, and the nucleoli are somewhat indistinct. In addition, elevated levels of mitosis and necrosis also form part of the histopathology examination.

The clinical presentation varies greatly, ranging from sore throat to hoarseness and dysphagia, typically coupled with a history of weight loss. In addition, unusual presentation with paraneoplastic syndrome due to an active hormone-secreting neuroendocrine tumor has also been reported [9]. However, our patient showed no such findings except a sore throat with a significant weight loss.

Primary small cell laryngeal cancer is sporadic. Therefore, no defined treatment guidelines are followed. Laryngeal small cell cancer management continues to prove difficult, and various modalities have been tried to conclude effective management strategies [10]. Surgery alone or in tandem with radiation has not shown effectiveness in local tumor control and is not recommended for initial management [7]. Similarly, although advantageous in local tumor control, isolated radiation management has been futile to improve mortality. However, combining primary radiation therapy with adjuvant chemotherapy has shown promising results with enhanced patient survival times [11]. Frequently used chemotherapeutic agents include cyclophosphamide, doxorubicin, vincristine, methotrexate, and lomustine [7]. Our patient underwent four cycles of carboplatin and etoposide, which improved the general condition initially until the relapsed disease was noted.

Consequently, the patient was started on combination therapy with nivolumab and ipilimumab. Luckily, a few weeks later, the patient achieved complete recovery. To date, six months post diagnosis, the patient is living a healthy life. Unfortunately, the prognosis remains poor despite aggressive management, with five-year survival at 19.3%. These considerably low prognostic figures are linked with the fact that greater than 90% of patients develop metastatic disease, with a significant portion of the patients presenting initially with metastatic disease [7].

## Conclusions

Laryngeal cancer of small cell neuroendocrine origin is a rare entity. As the tumor presents with clinical manifestations similar to other subtypes, histopathology is often required to diagnose. Small cell carcinoma of the larynx possesses aggressive properties, with many patients presenting with metastatic diseases. This cancer is rare, so the treatment modalities have not been experimented with very well. Studies show radiotherapy with adjuvant chemotherapy improves patient survival.

## References

[1] Siegel RL, Miller KD, Fuchs HE, Jemal A (2021). Cancer statistics, 2021. CA Cancer J Clin.

[2] Jaiswal VR, Hoang MP (2004). Primary combined squamous and small cell carcinoma of the larynx: a case report and review of the literature. Arch Pathol Lab Med.

[3] Olofsson J, Van Nostrand AW (1972). Anaplastic small cell carcinoma of larynx: case report. Ann Otol Rhinol Laryngol.

[4] Singh H, Chauhan A (2011). Primary small cell carcinoma of the larynx: report of a rare tumor. Case Rep Oncol Med.

[5] Perez-Ordoñez B (2018). Neuroendocrine carcinomas of the larynx and head and neck: challenges in classification and grading. Head Neck Pathol.

[6] Ferlito A, Devaney KO, Hunt JL, Hellquist H (2019). Some considerations on the WHO histological classification of laryngeal neoplasms. Adv Ther.

[7] Ferlito A, Silver CE, Bradford CR, Rinaldo A (2009). Neuroendocrine neoplasms of the larynx: an overview. Head Neck.

[8] Ferlito A, Rinaldo A (2003). Small cell neuroendocrine carcinoma of the larynx: a preventable and frustrating disease with a highly aggressive lethal behavior. ORL J Otorhinolaryngol Relat Spec.

[9] Thompson LD (2016). Neuroendocrine tumors of the larynx. Ear Nose Throat J.

[10] Strojan P, Hernandez-Prera JC, Beitler JJ (2019). Small cell and large cell neuroendocrine carcinoma of the larynx: a comparative analysis. Cancer Treat Rev.

[11] van der Laan TP, Plaat BE, van der Laan BF, Halmos GB (2015). Clinical recommendations on the treatment of neuroendocrine carcinoma of the larynx: a meta-analysis of 436 reported cases. Head Neck.

